# Transmission Dynamics of *Trichomonas tenax*: Host and Site Specificity, Zoonotic Potential, and Environmental Factors

**DOI:** 10.3390/microorganisms13071475

**Published:** 2025-06-25

**Authors:** Maurice Matthew, Jennifer Ketzis, Samson Mukaratirwa, Chaoqun Yao

**Affiliations:** One Health Center for Zoonoses and Tropical Infectious Diseases, Ross University School of Veterinary Medicine, P.O. Box 334, Basseterre KN0101, Saint Kitts and Nevis; mmatthew@rossvet.edu.kn (M.M.); jketzis@rossu.edu (J.K.); smukaratirwa@rossvet.edu.kn (S.M.)

**Keywords:** *Trichomonas tenax*, transmission, zoonosis, host, environmental factors

## Abstract

*Trichomonas tenax* is an anaerobic flagellate usually found in the oral cavity of humans and domestic animals. It is very likely to be transmitted through kissing, sharing saliva, contaminated utensils, and water. However, research on its transmission dynamics is scarce. Hence, there is a need to identify potential knowledge gaps in *T. tenax* transmission for future research and emphasize the importance of the One Health approach in controlling the spread of this flagellar protozoan. *Trichomonas tenax* has been found in humans, dogs, cats, horses, and birds at various body sites, including the lungs and the urogenital tract, in addition to the oral cavity. Its transmission is influenced by environmental factors such as temperature and socioeconomic factors such as age, income, smoking, and public awareness, along with poor oral hygiene and systemic diseases. Direct host-to-host transmission also plays an important role; however, transmission through fomites or contaminated water still needs to be scientifically proven to gain a better understanding of these mechanisms. More studies on this flagellate are warranted, especially using animal models and epidemiological studies, to better understand its transmission dynamics. Prioritizing research in these areas could result in a more comprehensive understanding of *T. tenax* transmission dynamics and the factors that influence it, ultimately aiding in the development of effective control and prevention strategies. It is also recommended to encourage collaboration between medical and veterinary professionals in addressing this zoonotic protozoan, recognizing that it aligns with the One Health approach.

## 1. Introduction

*Trichomonas tenax*, an aerobic protozoan, is commonly found in the oral cavities of humans and animals with inadequate oral hygiene and advanced periodontal disease, respectively [1,2]. This mitochondrion-lacking protist, which is closely related to *Trichomonas vaginalis*, is an ovoid or ellipsoidal flagellate measuring 5–16 × 2–15 µm in size [3,4]. Its prevalence ranges from 1 to 90% in humans and 8 to 96% in dogs [5]. Over the years, *T. tenax* has been strongly associated with periodontal disease [6,7,8]. This is one of the most common inflammatory diseases and public health issues in humans and animals worldwide [9]. The disease may lead to tooth loss and disability, negatively affect chewing function and aesthetics, and impair the quality of life [10]. Furthermore, *T. tenax* exhibits parasite-like behavior akin to *T. vaginalis*, so it should be considered a parasite rather than a commensal [11]. Moreover, *T. tenax* has been shown to release virulent proteinases, such as cysteine protease, further supporting its potential pathogenicity [12,13,14]. These proteases play a vital role in biological processes, including the nutrient absorption, invasion, virulence, and immune evasion of parasitic organisms [15].

While various transmission routes for *T. tenax* have been proposed, such as via saliva droplets, drinking water, kissing, or the use of contaminated dishes, there are still no definitive reports confirming these modes of transmission [16,17,18,19]. Recent advances in sensitive methods for the detection of *T. tenax* (e.g., PCR and LAMP) could enable better determination of transmission modes and prevalence [20,21,22].

A systematic review and meta-analysis found that the global pooled prevalence of *T*. *tenax* in humans is 17% (95% Confidence Interval 14–22%) [23]. Despite a well-established association between *T. tenax* and periodontal disease, the parasite remains greatly neglected in research and clinical attention [5,23]. To identify research gaps and future research directions, this article mainly addresses the transmission dynamics of *T. tenax*, including hosts, sites of infection, and factors that might contribute to its transmission. The specific questions included are as follows: (1) What are the known host species and predilection site(s) for *T. tenax*? (2) What factors contribute to its host specificity? (3) Are there documented cases of zoonotic transmission of *T. tenax*? (4) What environmental factors are associated with the transmission of *T. tenax*? (5) What gaps exist in the current understanding of *T. tenax* transmission dynamics? (6) What future research directions should be prioritized to address these gaps?

## 2. Hosts

### 2.1. Host and Site Specificity

Trichomonads exhibit a preference for specific hosts and sites. *Trichomonas tenax* is noted for the diversity of aberrant sites beyond the predilection site of the oral cavity and accidental infections in humans and other animals (Table 1) [24]. *Trichomonas tenax* was first reported in humans in the oral cavity by Muller in 1773 from aqueous solutions of tartar [25,26]. While humans and the oral cavity are considered the preferred host and site of infection, *T. tenax* has also been identified in the lungs of humans, causing pulmonary trichomoniasis [27,28,29,30,31], as well as the submaxillary gland [32]. In one case study, *T. tenax* was isolated from an 82-year-old patient with asthenia, showing a co-infection with *Mycobacterium tuberculosis,* after the lymph node was dissected and cultured [33]. Additionally, studies have unequivocally reported *T. tenax* in the urogenital tract of humans, suggesting its ability to invade this site along with its close relative, *T. vaginalis* [34,35]. In addition to diverse sites of infection in humans, *T. tenax* has been observed to infect diverse hosts. It has been found in the oral cavity of dogs, cats, and horses, with dogs being the most identified non-human host. It is unclear whether the higher prevalence in dogs is due to host characteristics or the number of studies with dog hosts compared to other potential non-human hosts [1,8,36,37]. In dogs, *T. tenax* has been found not only in the mouth but also, in one study, in the mandibular gland of a 13-year-old dog that had a swelling of the left submandibular region. After aspiration from the gland was examined, numerous numbers of *T. tenax* were discovered [38]. An additional trichomonad species *T. brixi* was found in dogs and cats in the Czech Republic, with a prevalence of 30.6% (34/111) and 6.6% (8/122), respectively. This was higher than the prevalence of *T. tenax* among these two hosts, which was 8.1% (9/111) and 4.1% (5/122) [37]. In regard to other aberrant infections, several studies have reported the presence of *T. tenax* in the cloaca of different types of birds along with *Trichomonas gallinae* [39,40,41]. This broad host range along with its ability to establish outside of the oral cavity suggests that *T. tenax* is potentially zoonotic and has been greatly overlooked.

The factors contributing to the host and oral cavity preference of *T. tenax* are not completely understood. However, potential influences such as the oral environment in which it thrives, host-specific receptors, and the immune response may play a pivotal role. In vitro culturing studies with *T. tenax* show that it thrives in an environment at a temperature of approximately 35 °C and in a pH range of 6.5–7.0. For a successful culture, it often requires serum (in most cases, horse serum, but bovine serum can be used as well) [20,47,48]. In terms of host-specific receptors, there is a strong possibility that there are specific receptors or surface molecules within the different hosts that *T. tenax* can adhere to and colonize like *T. vaginalis*. However, there have been no reported studies on the possibilities of this phenomenon to date. The immune response of the host species could be a key player in limiting the ability of *T. tenax* to cause infections. Hong et al. (2023) [19] explored the cytotoxic effects and immune effects of *T. tenax* on gum epithelial and pulmonary cell lines in vitro. They found that *T. tenax* induces a cytotoxic effect on gum epithelial cells by disrupting cellular junctions. However, it imposes little damage on pulmonary cells. *T. tenax* induces the production of IL-6 at a low multiplicity of infection in both cells [19]. The differences between host immune responses and cytotoxic effects with affinity to gum epithelial cells can give possible insights into host and site specificity.

### 2.2. Zoonotic Potential

*Trichomonas tenax* is not typically considered a zoonotic or anthropozoonotic pathogen; however, there have been some reports in the literature that support the potential of *T. tenax* to be passed to and from dogs and humans [1,37]. The first recorded report of *T. tenax* anthropozoonotic potential was in 1928 by H. Hinshaw, who tried to infect dogs with both *Entamoeba gingivalis* and *T. tenax* cultured from a human patient with periodontitis. Dogs with normal gingiva inoculated via direct contact did not become infected; however, human-isolated *T. tenax* and *E. ginigvalis* did establish in an old dog with advanced gingivitis. Furthermore, the pathological findings in the oral cavity were similar to those in human periodontitis at 14.5 months post-inoculation during necropsy after the dog was euthanized [49]. This experimental infection, although suboptimal in design, indicates that it takes many months from establishing infection by both protozoa to inducing pathological changes in the mouths of dogs. Nevertheless, the study was unable to show that the pathological changes were due to *T. tenax* infection alone. In a review on zoonotic trichomonads, Maritz et al. (2014) highlighted the wide host range of *T. tenax* as a concern that should be taken seriously and further investigated to assess its risk to human health [50]. Additionally, a study conducted in Poland identifying *T. tenax* in domesticated animals using PCR reported that for three dogs that were positive for *T. tenax*, their owners were also positive for *T. tenax*, therefore illustrating the possibility that oral trichomoniasis can spread between humans and domestic dogs [1]. Moreover, sequencing and phylogenetic analyses have been used to detect *T. tenax* in oral samples from dogs and cats, further proving that this parasite infecting the human oral cavity is capable of infecting dogs and cats and could possibly be transmitted between different species of hosts [37,38,51]. Other studies, with birds, have identified the presence of *T. tenax* by sequencing and phylogenetics. Together, these studies, in dogs, cats, and birds, strongly indicate zoonotic or anthropozoonotic potential and suggest that more attention on this neglected protozoan is needed by both medical and veterinary professionals [35,39,41].

## 3. Factors Affecting Transmission and Prevalence

### 3.1. Factors Affecting Transmission

Various factors can impact the transmission and prevalence of *T. tenax*, including environmental conditions such as temperature, pH levels, urban or rural environments, oral hygiene practices, the oral cavity micro-environment, and geographical distribution (Table 2) [20]. However, there is limited research on the effects of environmental factors on the transmission and prevalence of this oral flagellate. It is noteworthy that geographical distribution has no impact on the transmission or prevalence of *T. tenax*, as was demonstrated in two studies that examined the global prevalence and distribution of *T. tenax*. These reports revealed that *T. tenax* is found on all continents except Australia, with varying prevalence across Africa, America, Asia, and Europe [5,52]. Its presence or lack thereof in Australia is unclear due to the limited number of studies conducted. In general, the prevalence of *T. tenax* has not differed between rural and urban areas, with both types of areas having high and low prevalence of *T. tenax*, depending on the population assessed [2,42,43,53,54]. The results of one study, which did report a difference in rural and urban populations (18.9% vs. 81.2%), might have been influenced by differences in oral hygiene in these populations [44].

The primary factors that affect the presence and transmission of *T. tenax* are poor oral hygiene and changes in oral micro-environments [27,45], consequently resulting in a strong association with periodontal disease in both humans and animals [2,6,8,16]. Additionally, research has demonstrated how certain systemic diseases, such as Down syndrome, renal failure, and diabetes, impact the oral cavity and, in turn, influence both the prevalence and transmission of *T. tenax* [18,46,58]. Temperature and pH are also environmental factors that impact both the survival and prevalence of *T. tenax*. Studies have illustrated that the oral flagellate has an optimal temperature ranging from 35 to 37 °C and pH between 6.5 and 7 [20,47,48]. Similarly, the closely related *T. vaginalis* and *T. gallinae* are also affected by temperatures between 35 and 37 °C and pH ranging between 6 and 7 [59,60]. Although these flagellates reside inside the body of their host, changes in the internal environment of the host due to temperature or pH can significantly impact their growth and survival. Other factors, such as socioeconomic factors (age, sex, income, and smoking) and public awareness, can also influence the transmission and prevalence of *T. tenax.* A study conducted in Iraq on 230 individuals reported that smoker patients showed statistical significance for the presence of *T. tenax* compared to non-smokers, and patients 30 years and older showed a higher rate of infection than younger ones [44]. Another study involving 310 patients with periodontal disease and 310 healthy controls revealed that the age group between the ages 41 and 50 years exhibited a higher prevalence of *T. tenax* than any of the younger age groups, as well as that males showed a higher incidence (24.7%) than females (16.8%) [55]. In Poland, researchers conducted a study on awareness of periodontal disease, risk factors, and the connection between periodontal disease and general health. They found that patients having an insufficient level of knowledge was related to risk factors as well as the prevention of periodontal disease [57]. Nazir et al. 2020 evaluated global data on patients with periodontal disease in low-, middle-, and high-income countries and discovered that the distribution of periodontitis differed significantly in lower- (28.7%), lower-middle- (10%), upper-middle- (42.5%), and high-income countries (43.7%) (*p* = 0.04) [56]. *Trichomonas vaginalis* prevalence and transmission have also been linked to socioeconomic factors; studies have shown that individuals living in poverty or with a low income were more likely to be infected with *T. vaginalis* as opposed to higher-income earners, and active smokers were more likely to be infected compared to non-smokers [61,62]. Enhancing our understanding of the environmental and socioeconomic factors influencing *T. tenax* prevalence and transmission is crucial for developing effective strategies to control its spread and mitigate its impact on human and animal health.

### 3.2. Transmission

**Figure 1:**
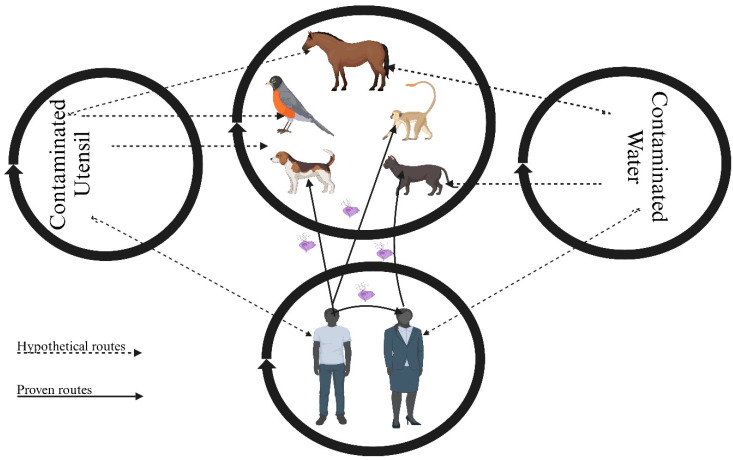
Schematic diagram showing the proven and hypothetical routes of *T. tenax* transmission. Created with https://BioRender.com.

Transmission is defined as the passing of an infectious agent from one host to another. Direct transmission routes include physical contact, contact with a contaminated environment or surface, airborne transmission, and fecal–oral transmission. Indirect transmission routes involve another organism, such as an insect vector or intermediate host [50]. It has been postulated that *T. tenax* can be transmitted through kissing, saliva, droplet spray, using contaminated dishes and utensils, and drinking contaminated water [16,17,63,64,65]. However, these modes of transmission are predominately extrapolated from what is known about other Trichomonads, with fewer experimental studies confirming that these modes of transmission are similarly suitable for *T. tenax*. In an experimental study performed in 1928, Hinshaw was able to directly infect a dog with *T. tenax*, which was obtained from a human mouth with advanced periodontal disease [49]. Other researchers after him have also been successful in directly infecting the mouths of monkeys, cats, and even humans with *T. tenax* [66]. In one study, a researcher in the 1930s inoculated *T. tenax* into his mouth and was able to establish an infection for a period of 10 months [67]. Similarly, *T. vaginalis* is normally transmitted via direct host-to-host contact, predominantly through sexual intercourse in humans [61,68,69,70]. One study also reported that *T. vaginalis* has been successfully transplanted into the vagina of monkeys but not in other mammals such as cows, horses, pigs, dogs, cats, rabbits, guinea pigs, rats, and woodrats [66]. *Trichomonas gallinae* is mainly transmitted through direct host-to-host transmission between parents and young birds during feeding [71]. One mechanism of transmission common among *T. tenax*, *T. vaginalis*, and *T. gallinae* is via direct host-to-host contact, which should be taken into consideration to mitigate the spread of these parasites. In terms of transmission through contaminated utensils, also known as fomite transmission, there were no recorded scientific studies conducted on *T. tenax* that illustrated that the oral flagellate can be transmitted by this means. Fomite transmission of *T. tenax* is possibly an assumption and is still not fully understood. The same goes for both *T. vaginalis* and *T. gallinae*; most studies assume or hypothesize that there is a strong possibility that these Trichomonads can be transmitted via fomites, but this too is not fully understood [69,72,73]. It has also been hypothesized that *T. tenax* can be transmitted via contaminated water. However, phylogenetic analyses based on the 16S ribosomal RNA (rRNA) gene suggest that Trichomonads are ancestral eukaryotic organisms [74]. According to M. Lopez and C Hall (2020), when eukaryotic cells are placed in water or hypotonic solutions, they get swollen and then rupture, a process known as cytolysis [75]. Additionally, based on cultural studies conducted on *T. tenax*, it has been shown that these Trichomonads require some form of serum for growth and survival [12,20,48]. Therefore, more studies on the transmission of *T. tenax* via contaminated water are needed to confirm this assumption (Figure 1). A study on the survival of *T. gallinae* in saline, water, and both dry and moist bird seeds was conducted; the study found that *T. gallinae* can survive for up to 168 h in saline and moist bird seeds but did not survive in water or dry bird seeds regardless of parasite density [72]. *Trichomonas vaginalis* has been reported to survive outside the human body in a wet environment for more than 3 h, suggesting that it can possibly be transmitted via contaminated water [73]. However, this mechanism of transmission is still not fully understood, and more studies are needed to confirm these hypotheses. The transmission of *T. tenax* involves several possible mechanisms within and between host populations; understanding these mechanisms can inform disease control strategies aimed at mitigating the spread of the parasite.

**Figure 2:**
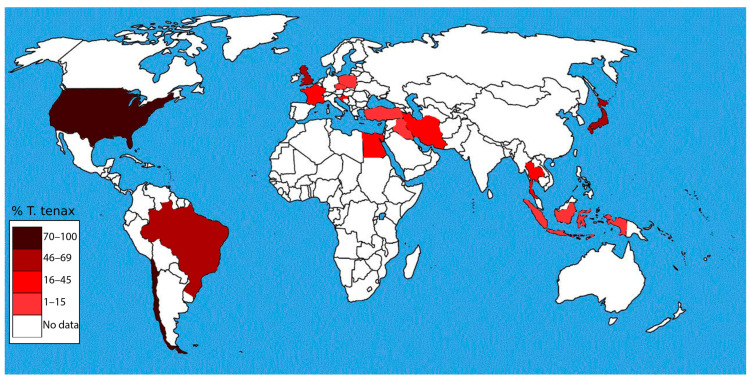
Color-coded world map highlighting regions where highest *T. tenax* prevalence in humans has been reported.

The mechanism by which *T. tenax* is transmitted is not fully understood, although there is evidence in humans that it can be transmitted through direct host-to-host contact, for example, through kissing, and saliva droplets, as shown in previous studies [49,67]. However, transmission through contaminated water or fomites is presently a suggestion and hypothesis that needs to be scientifically proven. More studies, especially animal model studies, are needed to gain a better and clearer understanding of the pathogenicity and transmission dynamics of *T. tenax*. Thus far, *T. tenax* has been reported on every continent except for Australia (Figure 2). Geographically, it is clear that location has no direct impact on the prevalence and transmission of *T. tenax*; however, socioeconomic factors, such as age, sex, income, and smoking for humans, and micro-environmental factors, such as temperature and pH, at the site of predilection may influence transmission (Table 2) [48,55]. Understanding the factors that influence the transmission of *T. tenax* is essential for developing effective prevention and control measures. These insights can inform targeted strategies aimed at reducing the spread of the parasite between humans and animals.

Knowledge of the transmission dynamics of *T. tenax* can also give valuable insights to researchers, policymakers, and public health authorities to develop and implement evidence-based control and prevention measures to counter the spread of *T. tenax*.

## 4. Future Research Prospects

The transmission dynamics of *T. tenax* are not fully understood. Studies that investigate the different routes of transmission, including water and food, as well as fomite contamination, are needed. Large-scale epidemiological studies, which are rendered practically feasible by the recent development of the LAMP diagnostic technique without the requirement of DNA extraction [22], are required to determine the prevalence, distribution, transmission dynamics, and risk factors associated with *T. tenax* infection in the various hosts where the parasite has been reported. Furthermore, public health education, especially for dog owners, that raises awareness on the importance of good oral hygiene and the potential for *T. tenax* transmission between humans and domestic animals is recommended. Prioritizing these research and educational areas could lead to more effective control and prevention strategies for *T. tenax* infections in the future.

## Figures and Tables

**Table 1:** Host(s) and infection site(s) in which *Trichomonas tenax* has been reported.

Host	Site	Type(s) of Study/Studies	Region(s)	Route of Transmission	Reference(s) *
Humans	Oral cavity	Cross-sectional, experimental, case–control	South America, Europe, Asia, Africa	Direct contact	[2,17,18,19,20,21,27,42,43,44,45,46]
Humans	Lung	Retrospective, case report	Europe, Asia	Unknown	[27,28,29,30]
Humans	Lymph node	Case report	Europe	Unknown	[33]
Humans	Submaxillary gland	Case report	Europe	Unknown	[32]
Humans	Urogenital tract	Systemic review, case report	Europe	Unknown	[34,35]
Dogs	Oral cavity	Cross-sectional	Europe, North America	Direct contact	[1,8,36,37]
Dogs	Mandibular gland	Case report	Europe	Unknown	[38]
Cats	Oral cavity	Cross-sectional	Europe	Direct contact	[1,37]
Horses	Oral cavity	Cross-sectional	Europe	Unknown	[1]
Birds	Cloaca	Cross-sectional	Asia, Europe	Unknown	[39,40,41]

* References are arranged in sequential order and align with the results presented in the table.

**Table 2:** Impacts of socioeconomic and micro-environmental factors on the transmission of *T. tenax* in humans.

Socioeconomic Factors				
**Age**	Older individuals	Older individuals tend to have weaker immune systems, making them more susceptible to *T. tenax* infection. Additionally, older individuals tend to have poor oral hygiene due to their inability to take care of themselves.	Iraq	[44,55]
**Sex**	Males	Males tend to have a higher prevalence than females, possibly due to poorer oral hygiene (females tend to care more about their oral hygiene than males) or hormonal influences (difference in hormones, e.g., testosterone vs. estrogen).	Iraq	[55]
**Income**	Limited access to dental care due to poverty Overpopulation and fast-paced lifestyles	Lower-income individuals do not have the financial resources to pay for oral hygiene care and, as a result, tend to have weaker immune systems and poor oral hygiene, increasing the risk of *T. tenax* infection. Higher-income individuals live in more populated areas and live busier lives, and as a result, they come into contact with more people and may neglect their oral hygiene due to being too occupied. This increases the risk of *T. tenax* infection.	Saudi Arabia	[56]
**Smoking**	Smokers	Smoking is deemed to weaken the host immune system and damage the oral cavity, thus increasing the risk of *T. tenax* infection and periodontal disease.	Iraq	[44]
**Public awareness**	Lack of oral hygiene awareness	A lack of education and awareness on the importance of good oral hygiene practices leads to poor practices and risky behavior, increasing the chances of *T. tenax* infection.	Poland	[57]
**Micro-Environmental Factors**				
**Temperature**	Warm environments	*Trichomonas tenax* reproduces best at 35 °C compared to other temperatures, which means that its growth is affected by temperature, which indirectly impacts its transmission.	Iran, Poland, Egypt	[18,46,58]
**pH**	Slightly neutral or neutral pH	*Trichomonas tenax* reproduces best at a pH of 6.5–7 compared to other pHs, which means its growth is affected by the pH of the environment, which indirectly impacts its transmission.	Iran, Poland, Egypt	[18,46,58]
**Oral hygiene practices**	Poor oral hygiene	Poor oral hygiene significantly impacts transmission since *T. tenax* thrives in conditions where the oral cavity is poorly maintained.	Poland, Chile	[1,2]
**Changes in oral micro-environments**	Acute or chronic	Changes in the oral micro-environment can either promote or inhibit the growth of *T. tenax*.	St. Kitts, France, Iran	[5,7,23]

## Data Availability

Data sharing is not applicable. No new data were created or analyzed in this study.

## References

[1] Dybicz M., Perkowski K., Baltaza W., Padzik M., Sędzikowska A., Chomicz L. (2018). Molecular identification of *Trichomonas tenax* in the oral environment of domesticated animals in Poland–Potential effects of host diversity for human health. Ann. Agric. Environ. Med..

[2] Bracamonte-Wolf C., Orrego P.R., Muñoz C., Herrera D., Bravo J., Gonzalez J., Varela H., Catalán A., Araya J.E. (2019). Observational cross-sectional study of *Trichomonas tenax* in patients with periodontal disease attending a Chilean University Dental Clinic. BMC Oral Health.

[3] Honigberg B.M., Lee J.J. (1959). Structure and division of *Trichomonas tenax* (O. F. Müller). Am. J. Epidemiol..

[4] Nagao E., Yamamoto A., Igarashi T., Goto N., Sasa R. (2000). Two distinct hemolysins in *Trichomonas tenax* ATCC 30207. Oral Microbiol. Immunol..

[5] Matthew M.A., Yang N., Ketzis J., Mukaratirwa S., Yao C. (2023). *Trichomonas tenax*: A neglected protozoan infection in the oral cavities of humans and dogs—A scoping review. Trop. Med. Infect. Dis..

[6] Marty M., Lemaitre M., Kémoun P., Morrier J.J., Monsarrat P. (2017). *Trichomonas tenax* and periodontal diseases: A concise review. Parasitology.

[7] Bisson C., Dridi S.M., Machouart M. (2019). Assessment of the role of *Trichomonas tenax* in the etiopathogenesis of human periodontitis: A systematic review. PLoS ONE.

[8] Patel N., Colyer A., Harris S., Holcombe L., Andrew P. (2017). The prevalence of canine oral protozoa and their association with periodontal disease. J. Eukaryot. Microbiol..

[9] Mohamad S., Mohamed Z. (2017). Chapter 4 Detection of *Trichomonas tenax* and *Entamoeba gingivalis*: A review. Updates in Parasitology.

[10] García-Huerta O.E., Chávez-Ruvalcaba F., Chávez-Ruvalcaba M.I., Chávez-Ruvalcaba K.M., Díaz-Alfaro L. (2012). Periodontal Diseases-A Clinician’s Guide.

[11] Ribeiro L.C., Santos C., Benchimol M. (2015). Is *Trichomonas tenax* a parasite or a commensal?. Protist.

[12] El Sibaei M.M., Abdel-Fattah N.S., Ahmed S.A., Abou-Seri H.M. (2012). Growth kinetics, antigen profiling, and proteinase activity of Egyptian *Trichomonas tenax* isolates derived from patients having oral infections. Exp. Parasitol..

[13] Yamamoto A., Asaga E., Nagao E., Igarashi T., Goto N. (2000). Characterization of the cathepsin B-like proteinases of *Trichomonas tenax* ATCC 30207. Oral Microbiol. Immunol..

[14] Bózner P., Demeš P. (1991). Cell-associated and extracellular proteolytic activity of an oral flagellate, *Trichomonas tenax*. Arch. Oral Biol..

[15] Yang N., Matthew M.A., Yao C. (2023). Roles of cysteine proteases in biology and pathogenesis of parasites. Microorganisms.

[16] Athari A., Soghandi L., Haghighi A., Kazemi B. (2007). Prevalence of oral trichomoniasis in patients with periodontitis and gingivitis using PCR and direct smear. Iran. J. Public Health.

[17] Mahdi N.K., al-Saeed A.T. (1993). *Trichomonas tenax* in Basrah, Iraq. J. Pak. Med. Assoc..

[18] Mehr A.K., Zarandi A., Anush K. (2015). Prevalence of oral *Trichomonas tenax* in periodontal lesions of down syndrome in Tabriz, Iran. J. Clin. Diagnostic Res..

[19] Hong Z.B., Lai Y.T., Chen C.H., Chen Y.J., Chen C.C., Lin W.C. (2023). *Trichomonas tenax* induces barrier defects and modulates the inflammatory cytotoxicity of gingival and pulmonary epithelial cells. Parasite.

[20] Diamond L.S., Bartgis I.L. (1962). Axenic cultivation of *Trichomonas tenax*, the oral flagellate of man I. establishment of cultures. J. Protozool..

[21] Kikuta N., Yamamoto A., Fukura K., Goto N. (1997). Specific and sensitive detection of *Trichomonas tenax* by the polymerase chain reaction. Lett. Appl. Microbiol..

[22] Matthew M.A., Christie J., Yang N., Yao C. (2022). A loop-mediated isothermal amplification (LAMP) assay specific to *Trichomonas tenax* is suitable for use at point-of-care. Microorganisms.

[23] Eslahi A.V., Olfatifar M., Abdoli A., Houshmand E., Johkool M.G., Zarabadipour M., Abadi P.A., Ghorbani A., Mirzadeh M., Badri M. (2021). The neglected role of *Trichomonas tenax* in oral diseases: A systematic review and meta-analysis. Acta Parasitol..

[24] Yao C., Ketzis J.K. (2018). Aberrant and accidental trichomonad flagellate infections: Rare or underdiagnosed?. Trans. R. Soc. Trop. Med. Hyg..

[25] Dobell C. (1939). The common flagellate of the human mouth, *Trichomonas tenax* (O.F.M.): Its discovery and its nomenclature. Parasitology.

[26] Goodey T., Wellings A.W. (1917). Observations on *Entamoeba gingivalis* from the human mouth, with a note on the trichomonad flagellate *Tetratrichomonas buccalis* n. sp. Parasitology.

[27] Hersh S.M. (1985). Pulmonary trichomoniasis and *Trichomonas tenax*. J. Med. Microbiol..

[28] Wu Y., Ye Y., Yang Y., Yang W., Lin J., Cao K. (2021). Pyopneumothorax from coinfection by *Trichomonas tenax* and *Geotrichum capitatum* in a child from China: A case report. BMC Infect. Dis..

[29] Cai D.H., Fang X.L. (2022). Pyopneumothorax caused by *Trichomonas tenax* and *Porphyromonas endodontalis* coinfection in a patient with previous cerebral infarction: A case report and literature review. Infect. Drug Resist..

[30] Mallat H., Podglajen I., Lavarde V., Mainardi J.L., Frappler J., Cornet M. (2004). Molecular characterization of *Trichomonas tenax* causing pulmonary infection. J. Clin. Microbiol..

[31] Duboucher C., Caby S., Chabé M., Gantois N., Delgado-Viscogliosi P., Pierce R., Capron M., Dei-Cas E., Viscogliosi É. (2007). Human pulmonary trichomonoses. Press. Medicale.

[32] Duboucher C., Mogenet M., Perie G. (1995). Salivary trichomoniasis: A case report of infestation of a submaxillary gland by *Trichomonas tenax*. Arch. Pathol. Lab. Med..

[33] Duboucher C., Farto-Bensasson F., Chéron M., Peltier J.Y., Beaufils F., Périé G. (2000). Lymph node infection by *Trichomonas tenax*: Report of a case with co-infection by *Mycobacterium tuberculosis*. Hum. Pathol..

[34] Fedorych P.V., Mavrov G.I., Osinska T.V., Shcherbakova Y.V. (2020). Protozoan genital invasions caused by the representatives of *Trichomonas* and *Giardia*. Wiad. Lek..

[35] Brosh-Nissimov T., Hindiyeh M., Azar R., Smollan G., Belausov N., Mandelboim M., Rahav G., Keller N., Gefen-Halevi S. (2019). A false-positive *Trichomonas vaginalis* result due to *Trichomonas tenax* presence in clinical specimens may reveal a possible *T. tenax* urogenital infection. Clin. Microbiol. Infect..

[36] Hegner R., Ratcliffe H. (1927). Trichomonads from the mouth of the dog. J. Parasitol..

[37] Kellerová P., Tachezy J. (2017). Zoonotic *Trichomonas tenax* and a new trichomonad species, *Trichomonas brixi* n. sp., from the oral cavities of dogs and cats. Int. J. Parasitol..

[38] Szczepaniak K., Łojszczyk-Szczepaniak A., Tomczuk K., Skrzypek T., Lisiak B., Abd-Al-Hammza Abbass Z. (2016). Canine *Trichomonas tenax* mandibular gland infestation. Acta Vet. Scand..

[39] Jiang X., Sun J., Wang F., Li H., Zhao X. (2016). Prevalence of *Trichomonas* spp. in domestic pigeons in Shandong Province, China, and genotyping by restriction fragment length polymorphism. Vet. J..

[40] Landman W.J.M., Gantois N., Sawant M., Majoor F.A., van Eck J.H.H., Viscogliosi E. (2021). Prevalence of trichomonads in the cloaca of wild wetland birds in the Netherlands. Avian Pathol..

[41] Martínez-Herrero M.C., Garijo-Toledo M.M., Liebhart D., Ganas P., Martínez-Díaz R.A., Ponce-Gordo F., Carrero-Ruiz A., Hess M., Gómez-Muñoz M.T. (2017). Novel avian oropharyngeal trichomonads isolated from European turtle doves (*Streptopelia turtur*) and racing pigeons (*Columba livia*): Genetic and morphometric characterisation of clonal cultures. Infect. Genet. Evol..

[42] Palmieri J.R., Halverson B.A., Sudjadi S.T., Purnomo, Masbar S. (1984). Parasites found in the mouths of inhabitants of three villages of South Kalimantan (Borneo), Indonesia. Trop. Geogr. Med..

[43] Ali Mohammed S.A., Mohsen Alwaaly A.B. (2019). Prevalence *Trichomonas tenax* in Karbala Governorate. J. Phys. Conf. Ser..

[44] Fadhil Ali Malaa S., Abd Ali Abd Aun Jwad B., Khalis Al-Masoudi H. (2022). Assessment of *Entamoeba gingivalis* and *Trichomonas tenax* in patients with chronic diseases and its correlation with some risk factors. Arch. Razi Inst..

[45] Norberg C.M.B.M. (2014). *Entamoeba gingivalis* (Gros, 1849) and *Trichomonas tenax* (Muller, 1773) oral infections in patients from Baixada Fluminense, Province of Rio de Janeiro, Brazil. Sci. J. Public Health.

[46] Dybicz M., Perkowski K., Sędzikowska A., Baltaza W., Chomicz L. (2018). Studies on prevalence of infection with *Trichomonas tenax* identified by molecular techniques–In respect to oral health of patients with various systemic disease requiring immunosuppressive therapy. Ann. Parasitol..

[47] Wantland W.W., Wantland E.M., Winquist D.L. (1963). Collection, identification, and cultivation of oral protozoa. J. Dent. Res..

[48] Asai S., Hayashi A., Nakamura Y., Kato M., Sato M., Nitta H., Namikawa I. (1986). Growth of *Trichomonas tenax* in tissue culture medium containing complement and antiserum to the accompanying bacteria. Jpn. J. Oral Biol..

[49] Hinshaw H.C. (1928). Experimental infection of dogs with *Endamoeba gingivalis* and *Trichomonas buccalis* of human mouth. Proc. Soc. Exp. Biol. Med..

[50] Maritz J.M., Land K.M., Carlton J.M., Hirt R.P. (2014). What is the importance of zoonotic trichomonads for human health?. Trends Parasitol..

[51] Ito N., Itoh N., Nakanishi R., Kameshima S., Kimura Y. (2023). Molecular prevalence and zoonotic potential of trichomonads from oral cavities in household dogs. J. Eukaryot. Microbiol..

[52] Stoyanov S., Tasinov O., Dimitrova T., Yaneva G. (2024). Prevalence of *Trichomonas tenax* in the population affected by periodontal disease—A review. Appl. Sci..

[53] Hinshaw H.C. (1926). Correlation of protozoan infections of human mouth with extent of certain lesions in pyorrhea alveolaris. Proc. Soc. Exp. Biol. Med..

[54] Ghabanchi J., Zibaei M., Afkar M.D., Sarbazie A.H. (2010). Prevalence of oral *Entamoeba gingivalis* and *Trichomonas tenax* in patients with periodontal disease and healthy population in Shiraz, Southern Iran. Indian J. Dent. Res..

[55] Al-Dulaimi F.H.A., Alajeely A.A.A., Ail Y.M. (2020). Incidence of *Entamoeba gingivalis* and *Trichomonas tenax* in periodontitis and gingivitis patients who Attended to private clincs in Babylon Province. Med.-Leg. Update.

[56] Nazir M., Al-Ansari A., Al-Khalifa K., Alhareky M., Gaffar B., Almas K. (2020). Global prevalence of periodontal disease and lack of its surveillance. Sci. World J..

[57] Dolińska E., Milewski R., Pietruska M.J., Gumińska K., Prysak N., Tarasewicz T., Janica M., Pietruska M. (2022). Periodontitis-related knowledge and its relationship with oral health behavior among adult patients seeking professional periodontal care. J. Clin. Med..

[58] Meabed E., Henin R. (2022). Prevalence of *Entamoeba gingivalis* and *Trichomonas tenax* among patients suffering from chronic systemic diseases in Egypt. Afro-Egypt. J. Infect. Endem. Dis..

[59] Kostara I., Carageorgiou H., Varonos D., Tzannetis S. (1998). Growth and survival of *Trichomonas vaginalis*. J. Med. Microbiol..

[60] Amin A., Neubauer C., Liebhart D., Grabensteiner E., Hess M. (2010). Axenization and optimization of in vitro growth of clonal cultures of *Tetratrichomonas gallinarum* and *Trichomonas gallinae*. Exp. Parasitol..

[61] Tompkins E.L., Beltran T.A., Gelner E.J., Farmer A.R. (2020). Prevalence and risk factors for *Trichomonas Vaginalis* infection among adults in the U.S., 2013–2014. PLoS ONE.

[62] Barbosa M.D.S., Andrade de Souza I.B., Schnaufer E.C.D.S., Silva L.F.d., Maymone Gonçalves C.C., Simionatto S., Marchioro S.B. (2020). Prevalence and factors associated with *Trichomonas vaginalis* infection in indigenous Brazilian women. PLoS ONE.

[63] Oladokun A.O., Opeodu O.I., Lawal A.O., Falade M.O. (2021). *Entamoeba gingivalis* and *Trichomonas tenax* in periodontal disease. Microbiol. Res. J. Int..

[64] Ibrahim S., Sc B., Sc M. (2012). Evaluation of *Entamoeba gingivalis* and *Trichomonas tenax* in patients with periodontitis and gingivitis and its correlation with some risk factors. J. Baghdad Coll. Dent..

[65] Al-Hasnawy M.H., Rabee A.H. (2023). A review on *Trichomonas* species infection in humans and animals in Iraq. Iraqi J. Vet. Sci..

[66] Wenrich D.H. (1947). The species of *Trichomonas* in man. J. Parasitol..

[67] Willcox R.R. (1960). Epidemiological aspects of human trichomoniasis. Br. J. Vener. Dis..

[68] Petrin D., Delgaty K., Bhatt R., Garber G. (1998). Clinical and microbiological aspects of *Trichomonas vaginalis*. Clin. Microbiol. Rev..

[69] Crucitti T., Jespers V., Mulenga C., Khondowe S., Vandepitte J., Buvé A. (2010). *Trichomonas vaginalis* is highly prevalent in adolescent girls, pregnant women, and commercial sex workers in Ndola, Zambia. Sex. Transm. Dis..

[70] Kissinger P. (2015). *Trichomonas vaginalis*: A review of epidemiologic, clinical and treatment issues. BMC Infect. Dis..

[71] Anderson N.L., Johnson C.K., Fender S., Heckly S., Metzler M., Nave P., Yim J. (2010). Clinical signs and histopathologic findings associated with a newly recognized protozoal disease (*Trichomonas gallinae*) in free-ranging house finches (*Carpodacus mexicanus*). J. Zoo Wildl. Med..

[72] McBurney S., Kelly-Clark W.K., Forzán M.J., Vanderstichel R., Teather K., Greenwood S.J. (2017). Persistence of *Trichomonas gallinae* in birdseed. Avian Dis..

[73] Crucitti T., Jespers V., Mulenga C., Khondowe S., Vandepitte J., Buvé A. (2011). Non-sexual transmission of *Trichomonas vaginalis* in adolescent girls attending school in Ndola, Zambia. PLoS ONE.

[74] Torres-Machorro A.L., Hernández R., Alderete J.F., López-Villaseñor I. (2009). Comparative analyses among the *Trichomonas vaginalis*, *Trichomonas tenax*, and *Tritrichomonas foetus* 5S ribosomal RNA genes. Curr. Genet..

[75] Lopez M.J., Hall C.A. (2020). Physiology, Osmosis.

